# Effectiveness and safety of elvitegravir/cobicistat/emtricitabine/tenofovir disoproxil fumarate single-tablet combination among HIV-infected patients in Turkey: results from a real world setting

**DOI:** 10.4314/ahs.v21i4.13

**Published:** 2021-12

**Authors:** Bilgul Mete, Alper Gunduz, Hayat Kumbasar Karaosmanoglu, Fatma Gumuser, Sibel Bolukcu, Dilek Sevgi Yildiz, Ozlem Altuntas Aydin, Bilgenur Bilge, Ilyas Dokmetas, Fehmi Tabak

**Affiliations:** 1 Istanbul University-Cerrahpasa, Cerrahpasa School of Medicine Department of Infectious Diseases and Clinical Microbiology; 2 Sisli Hamidiye Etfal Research and Training Hospital Department of Infectious Diseases and Clinical Microbiology; 3 Bakirkoy Dr Sadi Konuk Research and Training Hospital, Department of Infectious Diseases and Clinical Microbiology; 4 Goztepe Medeniyet University, Medical School Department of Infectious Diseases and Clinical Microbiology; 5 Bezm-i Alem University, Vakif Gureba Hospital, Department of Infectious Diseases and Clinical Microbiology; 6 Istanbul University-Cerrahpasa, Cerrahpasa School of Medicine Department of Medical Genetics All authors meet the ICMJE authorship criteria

**Keywords:** Elvitegravir/cobicistat/emtricitabine/tenofovir disoproxil fumarate, HIV, effectiveness, safety

## Abstract

**Background:**

Efficacy of elvitegravir/cobicistat/emtricitabine/tenofovir disoproxil (E/C/F/TDF) in treatment-naïve and experienced patients with HIV infection was demonstrated in phase 3 trials. The primary objective of this study was to evaluate effectiveness and safety of E/C/F/TDF in real world settings.

**Methods:**

Retrospective, observational data collected by the Turkish ACTHIV-IST study group between May 2015 and December 2016 were analysed.

**Results:**

A total of 387 patients were prescribed E/C/F/TDF; 210 patients with available data at 6th month were eligible; 91.5% were male, and mean age was 35.2 (SD: 10.8) years; 54.0% of males identified themselves as MSM. Sixty-three percent (133) of the study population were treatment-naïve patients, and 37% (77) were treatment experienced. HIV RNA level was below 100 copies/mL in 78.9% of treatment-naïve patients and 89.9% of treatment experienced patients at month 6. Median increase in CD4 T lymphocyte count was 218 copies/mL in treatment-naïve patients and remained stable or increased in treatment experienced patients. Adverse events were observed in 15% of the patients, and the regimen was discontinued in only six patients.

**Conclusion:**

Real world data on the effectiveness and safety of E/C/F/TDF is comparable with the phase 3 trial results Adverse events are uncommon and manageable.

## Introduction

The World Health Organisation (WHO) estimates that 36.7 million people were living with human immunodeficiency virus (HIV) in 2016, and 1.8 million of them were newly infected in that year. Moreover, HIV infection caused approximately 1 million death globally^1^. In 2016, 69.5% othe people living with HIV (PLWH) were diagnosed, of whom 76.5% were on antiretroviral therapy (ART). Of those on ART, 82.1% were virologically suppressed^1^.

The Turkish Public Health Authority reported that a total of 16644 people had been diagnosed with HIV infection from 1985 through June 2017, and 1537 people had been diagnosed with acquired immune deficiency syndrome (AIDS) during the same time period^2^. Although the exact number of alive PLWH in Turkey is not known, a recent study revealed that there may be 23192 adults living with HIV infection in Turkey, of whom half (48.9%) have been diagnosed^3^.

Since life-long treatment with an ART regimen consisting of at least three drugs is recommended, multi-tablet regimens (MTRs) may compromise adherence to treatment and result in decrease in quality of life and treatment success^4^. Single-tablet regimens (STRs) combing multiple agents are available and have been associated with clinically significant advantages over MTRs^5^.

The first boosted integrase strand transfer inhibitor (INSTI)-based combination available was a fixed combination of elvitegravir (150 mg), cobicistat (150 mg), emtricitabine (200 mg) and tenofovir disoproxil fumarate (245 mg) (E/C/F/TDF, Stribild®)^6^.

Efficacy of E/C/F/TDF in ART naïve and treatment experienced patients compared to other combinations was shown in phase trials. The results of the phase 3 trials revealed that E/C/F/TDF was effective in more than 80% of treatment-naïve and 78–87% of treatment experienced patients^7^–^11^.

E/C/F/TDF has been well tolerated by both treatment-naïve and treatment-experienced patients. Most adverse events reported in these studies were mild or moderate^7^–^17^. The most commonly reported adverse events (reported for ≥ 1/10) are abnormal dreams, asthenia, diarrhea, elevated creatine kinase, headache, hypophosphatemia, insomnia, nausea, rash and vomiting^6^. Patients on E/C/F/TDF should be followed for loss of bone mineral density and renal toxicity, including proximal tubulopathy. E/C/F/TDF is not recommended in patients with creatinine clearance < 70 mL/minute or previous renal impairment history due to TDF^6^.

E/C/F/TDF was approved in 2013 and has been widely used in Turkey. Real world data on the effectiveness and safety data of E/C/F/TDF has not been published in a detailed manner so far. We present real world data on the effectiveness and safety of E/C/F/TDF using one of the largest HIV databases in Turkey.

## Material and methods

ACTHIV-IST (ACTion against HIV in ISTanbul) is a study group founded in 2012. The aim of this local study group was to establish a database collecting data on PLWH from 5 major centres in Istanbul involving approximately 20% of HIV-infected patients in Turkey. The list of participating centres is included in the acknowledgement section.

Due to the observational nature of the study, data collection procedures of ACTHIV-IST database do not require interventions in addition to the routine practice of the centres; however, the centres are expected to record all available data in the database. Collected data include demographics, medical characteristics, HIV infection and treatment history, including adherence, HIV RNA levels, CD4 T-lymphocyte results, and adverse events. The primary objective of this analysis was to evaluate effectiveness and safety of E/C/F/TDF in real world settings in HIV-infected patients. Data collected from all treatment-naïve and -experienced patients on E/C/F/TDF who provided written consent and had at least 6 months of follow-up were included. There were no exclusion criteria. The data analysed in the manuscript were collected between May 2015 and December 2016. Each centre used its local laboratory, and each laboratory followed its own standard operating procedures. HIV RNA measurement technique varied across laboratories, and with one hospital using a detection threshold of 100 copies/mL. Therefore, an undetectable HIV RNA viral load was defined as < 100 copies/mL in this study.

Adverse events were classified and reported by using the medical dictionary for regulatory activities (Med-DRA).

No formal sample size calculation was made since all eligible patients were included using a consecutive sampling technique. Participating centres were expected to show maximum effort to minimize loss to follow-up. No replacement or adjustment analysis for missing data was planned for the patients lost to follow-up. Due to the nature of the study, descriptive statistics (count and percentage for categorical data; mean, standard deviation [SD]- or median, iter-quartile range [IQR]- for numeric data) were used to summarize the data. Analyses were performed intent-to-treat population. Some analyses were repeated for all treatment-experienced patients, treatment-experienced patients with a history of prior treatment failure patients and treatment-naïve patients with baseline HIV RNA level ≥100,000 copies/mL.

## Results

### Descriptive and medical characteristics

ACTHIV-IST database included data on 387 patients on E/C/F/TDF at the end of 2016 with 2736 patient-month follow-up data. Of those, 91.0% were male, mean age was 35.0 (SD: 10.7) years, and 53.4% of males described themselves as men who have sex with men (MSM). Of the 387 patients, 225 (58.1%) were ART naïve and 162 (41.9%) were antiretroviral treatment experienced (Table 1).

**Table 1:** Demographics and medical characteristics of patients in the ACTHIV-IST database

	Total (n=387)	ART experienced (n=162)	ART naive (n=225)
**Demographics**			
Gender, male; n (%)	352 (91.0%)	137 (84.6%)	215 (95.6%)
Age, years; mean (SD)	35.0 (10.7)	36.8 (11.0)	33.7 (10.3)
Sexual orientation, MSM; n (%)*	188 (53.4%)	114 (53.0%)	74 (54.0%)
**Medical characteristics**			
Positive HBsAg; n (%)	17 (4.4%)	9 (5.6%)	8 (3.6%)
Positive anti-HCV; n (%)	4 (1.0%)	1 (0.6%)	3 (1.3%)
Concomitant disease; n (%)**			
Any	126 (32.6%)	56 (34.6%)	70 (31.1%)
HPV infection	26 (6.7%)	8 (4.9%)	18 (8%)
Syphilis	22 (5.7%)	9 (5.6%)	13 (5.8%)
Diabetes mellitus	14 (3.6%)	4 (2.5%)	10 (4.4%)
Hypertension	14 (3.6%)	6 (3.7%)	8 (3.6%)
**HIV infection related parameters**			
HIV infection duration, month; median (IQR)	22.0(15.0–39.0)	39.5 (29.0–63.0)	16.0 (12.0–21.0)
Total antiretroviral treatment duration, month; median (IQR)	15.0 (10.0–28.0)	31.5 (23.0–54.0)	11.0 (8.0–14.0)
E/C/F/TDF treatment duration, month; median (IQR)	12.0 (8.0–14.0)	12.0 (8.0–15.0)	11.0 (8.0–14.0)
Patient received antiretroviral treatment within ≤3 months after HIV infection diagnosis; n (%)	218 (56.3%)	94 (58.0%)	124 (55.1%)

ART: Antiretroviral treatment E/C/F/TDF: elvitegravir/cobicistat/emtricitabine/tenofovir disoproxil fumarate, HPV: Human papillomavirus, IQR: inter-quartile range, MSM: men who have sex with men, SD: standard deviation

* Percentages are given in total male number

** disease with >1% listed only

Most of the treatment-naïve patients (95.6%) were male, with mean age 33.7 (SD: 10.3) years; 54.0% of males described themselves as MSM. HBsAg (3.6%) and anti-HCV (1.3%) positivity were uncommon, and one-third of patients (31.1%) had at least one comorbidity. Treatment-naïve patients were diagnosed with HIV infection for a median duration of 16.0 months (IQR: 12.0–21.0) and were on E/C/F/TDF for a median of 11.0 months (IQR: 8.0–14.0). E/C/F/TDF was started within 3 months of HIV diagnosis in 55.1% of treatment-naïve patients (Table 1).

Of the 162 treatment-experienced patients, 84.6% were male and mean age was 36.8 (SD:11.0) years; 53.0% of males described themselves as MSM. HBsAg (5.6%) and anti-HCV (0.6%) positivity were uncommon, and one-third of patients (32.6%) had at least one comorbidity. Treatment-experienced patients were diagnosed with HIV infection for a median duration of 39.5 months (IQR:29.0–63.0), had been on ART for a median of 31.5 months (IQR:23.0–54.0), and on E/C/F/TDF for a median of 12.0 months (IOR:8.0–15.0). ART was started within 3 months of HIV diagnosis in 58.0% of treatment-experienced patients. The most commonly used previous NRTI backbone was F/TDF (96.3%); the three most commonly used regimens were F/TDF + lopinavir/ritonavir (46.9%), F/TDF + efavirenz (43.2%), and F/TDF + darunavir + ritonavir (4.3%). Of the treatment-experienced patients; 24.1% (39/162) were switched to E/C/F/TDF due to treatment failure, and 75.9% (123/162) changed treatment to simplify the treatment (Table 1).

To be able to determine the real world safety and effectiveness of E/C/F/TDF, only the patients with available data at 6 months were evaluated in this study. A total of 210 patients were eligible, of whom 91.5% were male and mean age was 35.2 years (SD 10.8), and 54.0% of males identified themselves as MSM. Sixty-three percent (133) of the study population were treatment-naïve patients, and 37% (77) were treatment experienced

### Treatment-naïve patients

#### Virologic efficacy

**Figure 1:**
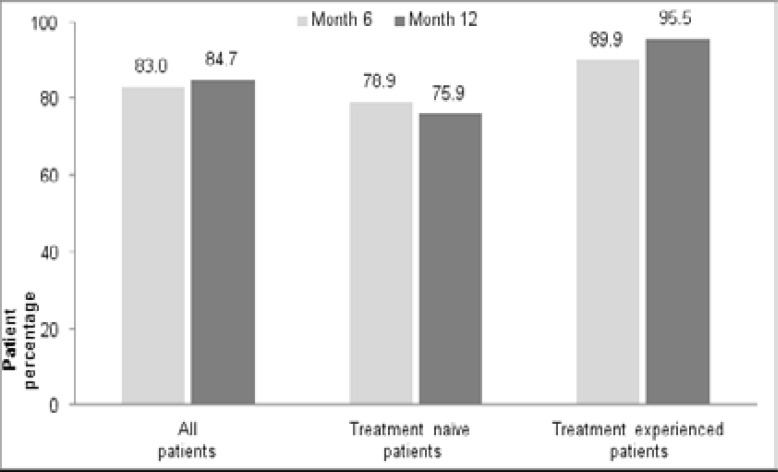
Percentage of patients with HIV RNA < 100 copies/ml at month 6 and month 12

**Figure 2:**
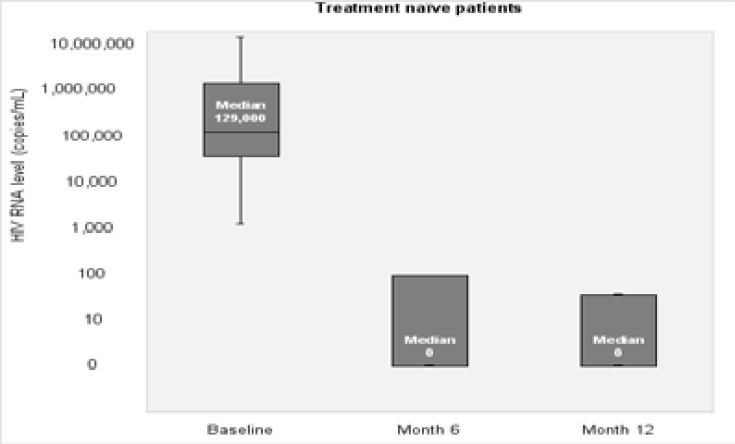
HIV RNA level (copies/mL) over time in treatment naïve patients

**Figure 3:**
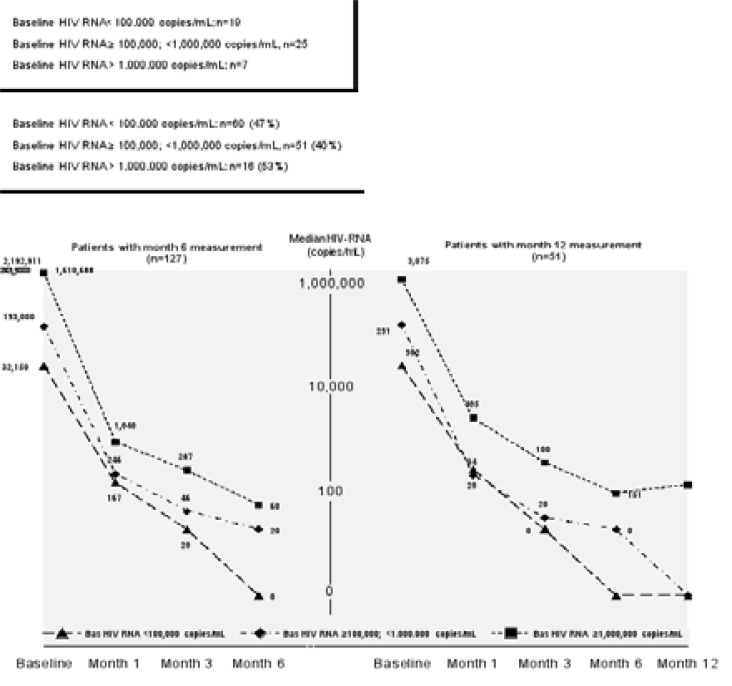
HIV RNA level (median, copies/mL) of patients with 6th month and 12th month measurements regarding baseline HIV RNA level subgroups in treatment naïve patients

Median baseline HIV-RNA level in treatment-naive patients was 112,228 copies/mL (IQR: 32,900–311,580). Six-month HIV RNA levels were available in 95% (127), and 38% (51) patients had available HIV RNA results at 12 months. HIV RNA level was below 100 copies/mL in 78.9% of treatment-naïve patients at month 6 (Figure 1). The patients were sub-grouped according to baseline HIV-RNA level as group I: < 100,000 copies/mL (47%), group II: 100,000 to <1,000,000 copies/mL (40%), and group 3: ≥1,000,000 copies/mL (13%). Median HIV-RNA level in all treatment-naïve patients decreased to 0 copies/mL (IQR: 0–93) at month 6. Median HIV-RNA level was 60 (IQR: 0–132) and 20 (IQR: 0–117) copies/mL in group III and group II, respectively. In 51 patients having results of HIV RNA level at month 12, median HIV RNA level was <100 copies/mL (Figure 2,3).

#### Immunologic recovery

Six-month CD4 T lymphocyte results were available in all of the patients (133) and in in 37% (49) at 12 months. Median CD4 T lymphocyte level in all treatment-naïve patients was 384 (IQR: 281–516) copies/mL at baseline and increased to 602 (IQR: 444–745) copies/mL at month 6 (median increase 218 copies/mL).

Patients were sub-grouped according to CD4 T lymphocyte level as group I: < 350 copies/mL (n=50), group II: 350 to <500 copies/mL (n=51), and group 3: ≥500 copies/mL (n=32).

**Figure 4:**
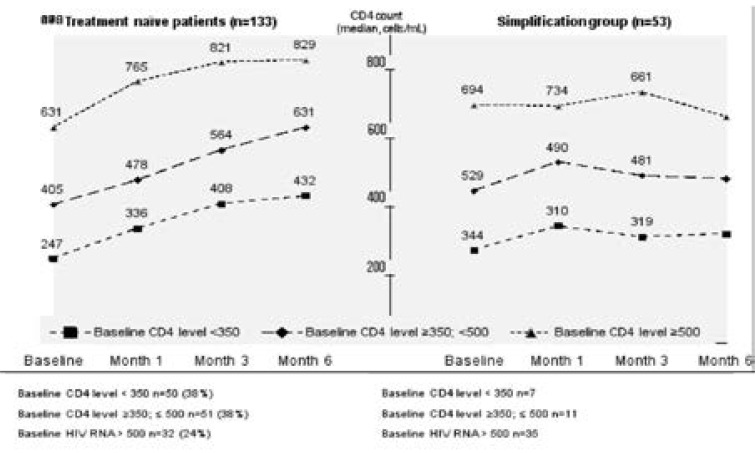
CD4+ T lymphocyte count (median, cells/mL) of patients with 6th month measurements regarding baseline CD4+ T lymphocyte level subgroups in treatment naïve patients (n=133) and samplification group (n=53)

Median CD4 T lymphocyte level was 247, 405 and 601 copies/mL at baseline and increased to 432, 631 and 829 copies/mL at month 6 in group I, II and III, respectively. Median CD4 T lymphocyte level continued to increase to 539 copies/mL in group I and remained stable in groups II and III at month 12 (Figure 4).

### Treatment experienced patients

#### Virologic efficacy

**Figure 5:**
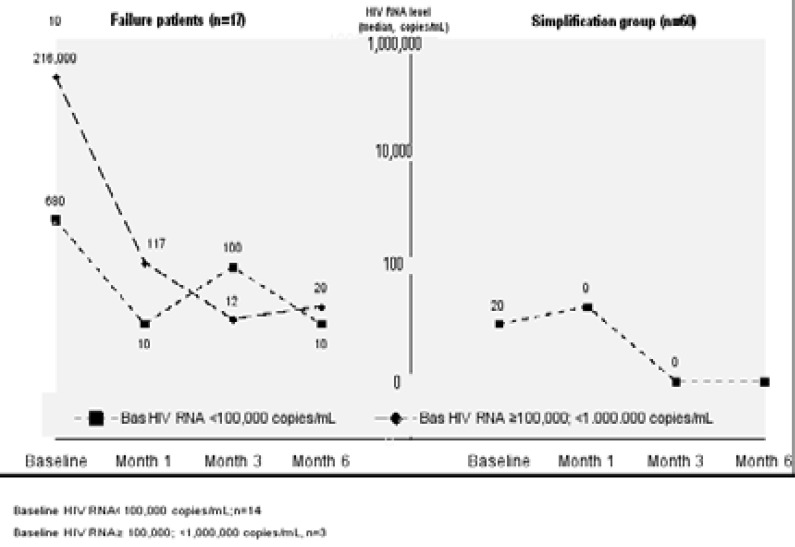
HIV RNA level (median, copies/mL) of patients with 6th month measurements regarding baseline HIV RNA level subgroups in treatment experienced (virological failure, n=17) and treatment experienced (simplification, n=60) patients

Median HIV-RNA level of all treatment experienced patients (n=77) was available at baseline and at 6th month. The patients were switched to E/C/F/TDF due to simplification in 60 patients and because of virologic failure in 17. Median HIV-RNA level of all treatment experienced patients was 20 (IQR: 0–72) and 0 (IQR: 0–20) copies/mL before E/C/F/TDF and at month 6, respectively, whereas HIV RNA level was below 100 copies/mL in 77.9 and 89.9% of all treatment experienced patients at before E/C/F/TDF and month 6, respectively (Figure 1). Median HIV RNA level was 10 (IQR: 0–20) and 0 (IQR: 0–20) copies/mL in the simplification group and 822 (IQR: 461–64,700) and 20 (IQR: 0–58) copies/mL in the virologic failure group before E/C/F/TDF and at month 6, respectively (Figure 5).

#### Immunological recovery

Median CD4 T lymphocyte level in treatment experienced patients before E/C/F/TDF and at month 6 was 611 (IQR:458–748) and 598 (IQR: 454–824) copies/mL, respectively. Since the number of available results was too small, 12-month data were not evaluated.

Due to the greater number of the patients, the increase in CD4 T lymphocyte count was evaluated primarily in the simplification group. The patients were subgrouped according to CD4 T lymphocyte level as group I: < 350 copies/mL (n=7), group II: 350 to <500 copies/mL (n=11), and group 3: ≥500 copies/mL (n=35) (Figure 4).

Median CD4 T lymphocyte level was 272, 446 and 696 copies/mL at baseline and increased to 319, 481 and 661 copies/mL at month 6 in group I, II and III respectively.

Seventeen patients were included in virologic failure group. Median CD4 T lymphocyte levels were 143, 451 and 652 copies/mL at baseline and increased to 234, 560 and 839 copies/mL at month 6 in group I, II and III, respectively.

#### Adverse events

Adverse events were analysed in 387 patients having withat least 6 months of follow-up. During E/C/F/TDF treatment, 14.9% (58) patients experienced adverse events. The adverse events (reported for >1.0% of patients) were nausea (8, 2.1%), diarrhea (7, 1.8%), pruritus (6, 1.6%), asthenia (5, 1.3%), headache (5, 1.3%), dizziness (4, 1.0%) and dyspepsia (4, 1.0%) (Table 2).

**Table 2:** Adverse events

Adverse event	n (% in 387)
Nausea	8 (2.1%)
Diarrhea	7 (1.8%)
Pruritus	6 (1.6%)
Asthenia	5 (1.3%)
Headache	5 (1.3%)
Dizziness	4 (1.0%)
Dyspepsia	4 (1.0%)
Flatulence	3 (0.8%)
Hyperhidrosis	3 (0.8%)
Somnolence	3 (0.8%)
Abdominal pain	2 (0.5%)
Alopecia	2 (0.5%)
Death	2 (0.5%)
Rash	2 (0.5%)
Weight increased	2 (0.5%)
**Total**	**58 (14.9%)**

Adverse events with incidence ≥%0.5 were listed in the table

Two patients (0.5%) died during treatment; neither death was attributed to E/C/F/TDF. Of 387 patients, six (1.6%) drug discontinuations were reported: due to death 2, drug-drug interaction 1, elevated creatinine 1, elevated liver enzyme 1, and physician decision 1.

## Discussion

INSTIs prevent or inhibit the binding of the preintegration complex to host cell DNA. INSTI-based regimens result in a rapid early-phase decay of plasma HIV-RNA. All currently available INSTIs are included now among the recommended regimens, and once-daily regimens are recommended for both treatment-naïve patients beginning ART and experienced patients receiving complex or poorly tolerated regimens and to use fixed-dose combinations (FDCs) and STRs when possible to decrease pill burden^13^.

E/C/F/TDF was approved by the U.S. Food and Drug Administration (FDA) in August 2012 in a once-a-day STR to treat ART-naïve patients or ART-experienced patients with no resistance to its components^14^.

Except for phase 3 studies and case reports, there is only one study evaluating the effectiveness and safety of E/C/F/TDF in the English literature so far^15^. This is the second study reporting real world data of the effectiveness of E/C/F/TDF in PLWH.

The results of the phase 3 trials revealed that E/C/F/TDF was effective in more than 80% of treatment-naïve and 78–87% of treatment experienced patients^7^–^11^.

In a phase 2 study comparing E/C/F/TDF to efavirenz (EFV)/F/ TDF for initial therapy, 90% of patients using E/C/F/TDF had HIV-1 RNA < 50 copies/mL at week 24 and 48, and a median increase of CD4 T-lymphocyte of 205 copies/mL was observed at the end of 48 weeks^12^.

A phase 3 study comparing E/C/F/TDF to EFV/FTDF revealed that 86.7% of treatment-naïve patients using E/C/F/TDF had HIV RNA < 50 copies/mL at week 48, and a median increase of CD4 T-lymphocyte of 239 copies/mL was observed after 48 weeks of treatment^8^. The long-term extension of this study revealed that viral suppression persisted; 84.2% and 80.2% of the patients had HIV-1 RNA < 50 copies/mL at week 96 and 144, respectively^7^,^8^.

In a phase 3 (study 103, compared to atazanavir + ritonavir + F/TDF); E/C/F/TDF 89.5% of participants were suppressed at week 48, and 83.3% and 77.6% of patients maintained suppression at weeks 96 and 144, respectively^16^–^18^. A median increase of CD4 T-lymphocyte of 207 copies/mL at 48 weeks was reported^16^.

In the present study HIV RNA suppression to below 100 copies/mL was observed in 78.9% of the treatment-naïve patients at 24 weeks. When compared to the phase 2/3 studies, rates of virological suppression similar, but it should be emphasized that HIV RNA level was ≥ 1,00,000 copies/ml in 13% and ≥ 100.000 copies/ml in 40% in our study. Squillace and colleagues found that viral load was undetectable in 77% of their naive patients (n=78) at 24 weeks, similar to our results^15^.

Median 6-month increase in CD4 T lymphocyte count was 218 copies/mL in our study, which is similar to phase 2/3 studies results demonstrating median increase of 205 to 239 copies/mL at 12 months.

Eighteen patients had HIV RNA >100 copies/mL at 24 weeks. When these patients were evaluated separately, 55% had baseline HIV RNA > 100,000 copies/ml, and 75% of the remaining patients were nonadherent to ART.

Adverse events, treatment failure, greater pill burden, and more frequent dosing have been associated with early ART discontinuation or modification^14^. The use of simpler and better tolerated regimens can improve outcomes. The efficacy of E/C/F/TDF in simplification strategy was evaluated in several trials. In the STRATEGY-PI study a phase 3b study switching from a ritonavir-boosted protease inhibitor plus F/TDF, 93.8% and 86.9% of patients had HIV-1 RNA < 50 copies/mL at week 48 and 96 by switching to E/C/F/TDF^14^,^16^. In the STRATEGY-NNRTI study, a phase 3b study switching from a non-nucleoside reverse transcriptase inhibitor (NNRTI) plus F/TDF, 93.4% and 86.6% of the patients switching to E/C/F/TDF had HIV-1 RNA < 50 copies/mL at week 48 and 96, respectively^10^–^11^.

Similar to the phase 3 trials in our study HIV RNA level remained below 100 copies/mL in approximately 90% of the all treatment experienced patients at month 6, and CD4 T-lymphocyte levels remained stable or increased depending on the level of CD4 cells.

E/C/F/TDF is well tolerated; the most common adverse events reported in registration trials were diarrhea, nausea, upper respiratory infection, and headache^14^. Studies by Derrick et al. valuated the safety profile of E/C/F/TDF and found that the most common adverse effect were primarily gastrointestinal (9%) followed by fatigue, headache, and skin manifestations. Overall, 20 (7%) patients discontinued therapy: five due to fatigue, gastrointestinal complications, and dizziness, five due to virologic failure with documented integrase resistance, three due to acute kidney injury, two due to economic burden, and five for undefined reasons^19^.

In our study approximately 15% patients experienced adverse events. The most commonly reported adverse effects were nausea (2.1%), diarrhea (1.8%), pruritus (1.6%), asthenia, headache, dizziness and dyspepsia. The discontinution rate was low (1.6%): two patients died due to reasons not attributed toE/C/F/TDF; four other discontinuations were reported due to drug-drug interactions, elevated creatinine, elevated liver enzyme, and physician decision.

Tenofovir alafenamide (TAF) was first approved by FDA in the form of the STR E/C/F/TAF (Genvoya ®) November 2015^20^. It has been a recommended regimen in the USA and European guidelines since 2015^4^,^21^. Phase 3 trials demonstrated that E/C/F/TAF was superior to E/C/F/TDF in terms of virologic efficacy in ART naïve and experienced patients due to lower rates of discontinuation of E/C/F/TAF compared with E/C/F/TDF^22^,^23^. However, E/C/F/TAF is not available in many countries in the world; therefore, real world data on E/C/F/TDF is and may also help to predict the real world effectiveness of E/C/F/TAF.

## Conclusion

Real world data on effectiveness and safety of E/C/F/TDF is comparable with the phase 3 trial results in both treatment-naïve and experienced patients. Adverse events are uncommon and manageable, and discontinuation rates are low. Since E/C/F/TAF and other newer regimens are not available worldwide, E/C/F/TDF may remain be recommended in the first-line regimens in treatment-naïve and experienced patients.
